# Iatrogenic salivary duct injury in head and neck cancer patients: Report of four cases and review of the literature

**DOI:** 10.4317/jced.51438

**Published:** 2014-07-01

**Authors:** Alena Kulyapina, Javier Lopez-de-Atalaya, Santiago Ochandiano-Caicoya, Carlos Navarro-Cuellar, Carlos Navarro-Vila

**Affiliations:** 1 Resident Physician in Oral and Maxillofacial Surgery. Department of Oral and Maxillofacial Surgery. Gregorio Marañon, General University Hospital. Madrid. Spain; 2 MD, PhD. Department of Oral and Maxillofacial Surgery. Gregorio Marañon, General University Hospital. Madrid. Spain; 3 MD. Department of Oral and Maxillofacial Surgery. Gregorio Marañon, General University Hospital. Madrid. Spain

## Abstract

Introduction: The lesions of the salivary ducts may be idiopathic, post- traumatic, or iatrogenic and lead to sialocele formation with persistent painful facial swelling or cutaneous fistula formation. No consensus on treatment of this condition exists: the options of treatment include needle aspiration, pressure dressings, antisialogogue therapy, radiotherapy, botulinum toxin and surgical approaches as duct repair, diversion, ligation, different drainage systems and even parotidectomy/submaxilectomy. The management and special features of iatrogenic salivary duct injury in patients with oral cancer who underwent head and neck reconstructive surgery has not been described yet. Material and Methods: We present four cases of iatrogenic lesions of salivary ducts and its management in patients with oral cancer. Conclusions: The iatrogenic lesions of salivary ducts are to be taken into account in patients with oral cancer as the distal ends of salivary ducts could be involved in the margins of surgical resection. Different options of treatment of this complication are described.

** Key words:**Sialocele, oral cancer, salivary duct.

## Introduction

Salivary duct injury may be post- traumatic or iatrogenic (1). A continuous salivary secretion without proper drainage leads to extravasation of saliva into the glandular or periglandular tissues and sialocele formation (2). Untreated sialocele may result in persistent painful facial swelling and cutaneous fistula formation (3). There is no consensus regarding the management of sialocele. There have been described different approaches such as percutaneous needle aspiration, pressure dressings, antisialogogue therapy, or radiotherapy, botulinum toxin and surgical approaches as duct repair, diversion, ligation, different drainage systems and even parotidectomy/submaxilectomy (4,5,6). The skin incision and drainage are often unsuccessful because of the recurrence of accumulation of fluid (1). There has not been specific reports about characteristic features and treatment of salivary duct surgical trauma in patients with oral cancer. This report examines the management of four cases of iatrogenic salivary duct injury in patients with oral cancer who underwent head and neck reconstructive surgery.

## Case 1

The patient, 54 year old female, was diagnosed for cancer of oral mucosa. She presented leucoplasic lesion of 1.5 cm of right buccal mucosa on the level of first upper molar. The squamous cell carcinoma diagnosis has been confirmed by biopsy. The patient underwent resection of lesion in oral mucosa with safety margins and unilateral modified neck dissection. The intraoral defect was reconstructed with radial forearm flap. The patients recovery was uneventful until the seventh postoperative day when she started with complaints of oedema and pain in the right parotid region where a fluctuating mass of 4 cm in the right parotid region was evidenced. The distal part of the Stensen’s duct was resected at the surgery and the radial forearm flap replaced the right buccal mucosa at the presumptive level of its opening. The urgent CT was performed which revealed Stensen’s duct dilation with presence of radial flap obstructing its distal end . The presumptive diagnosis of iatrogenic sialocele was done. The swelling persisted three days and resolved spontaneously with intraoral fistulization of the sialocele. No complications for radial forearm flap were produced. No recurrence of sialocele was observed during one year of postoperative follow up.

## Case 2

**Figure 1:**
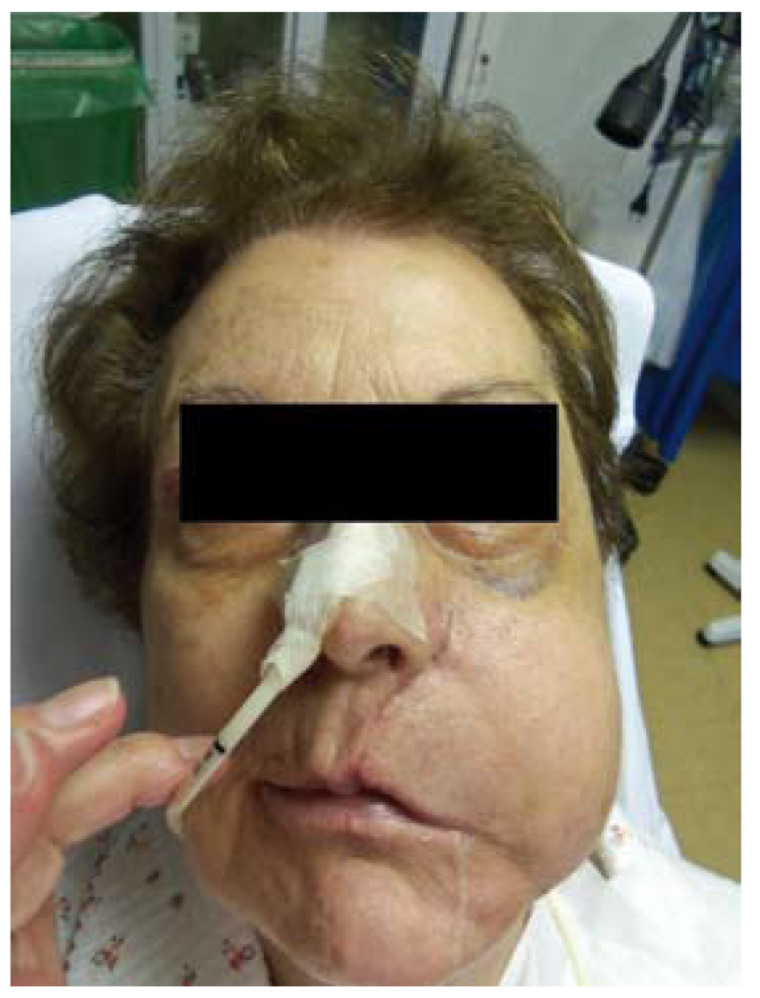
Patient developed left parotid sialocele on the sixth day after oral mucosa cancer resection.

The patient, 78 year old female, was diagnosed for recurrence of oral mucosa carcinoma. The patient was diagnosed for squamous cell carcinoma of the left tongue seven years before when she underwent resection of the tumour with marginal mandibulectomy and unilateral modified neck dissection. The surgical margins were clear and the patient was followed up. Three years after the recurrence of the squamous cell carcinoma in the left mandibular gingiva and left remanent tongue was detected. The patient underwent left mandibulectomy with reconstruction with fibular flap. Three years later the recurrence of squamous cell carcinoma was detected in the union of fibular flap and buccal mucosa. The patient underwent resection of lesion in oral mucosa with safety margins in block with posterior maxillectomy. The intraoral defect was reconstructed with temporal flap. The patient presented no complications in the early postoperative period. On the sixth day of the postoperative period a fluctuating mass of 5 cm in the left parotid region appeared (Fig. 1). The percutaneous puncture of the mass was performed obtaining 40 cl of yellow liquid. On the next day the recurrence of fluctuating mass was observed. The biochemical analisis of the liquid obtained on puncture showed the level of amylase of 180000 IU/l. The distal end of the Stensen’s duct was resected at the time of surgery thus the diagnosis of iatrogenic sialocele was made. The puncture of the mass was performed transorally with over- the- needle cannula, 40 cl of liquid was obtained and the facial swelling was resolved. The intraoral cannula was fixed with non- resorabable stitches. No recurrence of sialocele was observed after placement of cannula. The salivation was observed via cannula. The cannula was maintained during four weeks. After the retirement of cannula the intraoral salivary fistula formation was observed. The patient did not present the recurrence of sialocele during the three months follow up.

## Case 3

**Figure 2:**
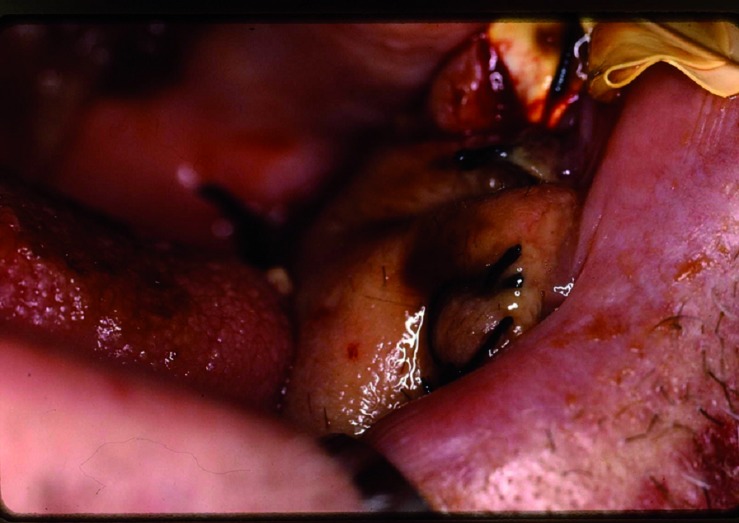
Intraoral view of rubber drain placed above radial forearm flap.

**Figure 3:**
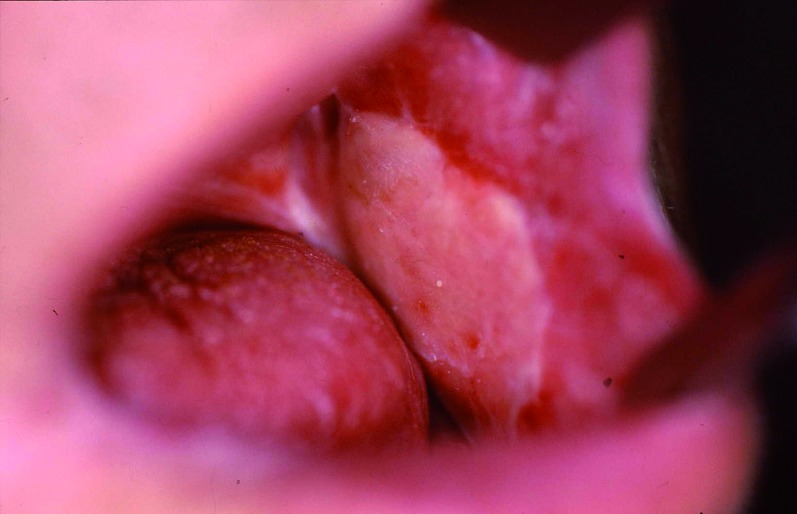
The permanent intraoral parotid fistula formation observed above radial forearm flap.

The patient, 55 year old male, presented lesion in the oral mucosa less than 1 cm from Stensen’s duct orifice. He was diagnosed for squamous cell carcinoma of oral mucosa. The oral mucosa was resected in block with the distal end of Stensen’s duct. The defect was repaired with radial forearm flap. A rubber drain was placed intraorally above the radial forearm island and fixed with stitches (Fig. 2). In the postoperative period the saliva flow was produced via rubber drain and no sialocele formation was evidenced. The drain was removed four weeks after surgery. The permanent intraoral parotid fistula formation was observed (Fig. 3).

## Case 4

The patient, 59 year old male, presented lesion in the mucosa of the floor of the mouth. The biopsy confirmed squamous cell carcinoma of oral mucosa. Transoral laser resection was performed. In the postoperative period the swelling in left submandibular region appeared. The CT revealed left submandibular sialocele formation. The pathology reported the margins of the specimenm of resected oral mucosa affected by the squamous cell carcinoma, thus the extension of surgical margins with bilateral selective neck dissection involving submandibular gland with dependent sialocele has been performed.

## Discussion

A number of papers exist about post traumatic silaocele. Iatrogenic sialocele have been described as complication of parotid surgery (7). There is no specific data about the cases of sialocele and salivary duct surgical injury in oral cancer patients. The aim of this study was to describe the cases of iatrogenic injury to the salivary duct and sialocele formation observed in postoperative period of oral cancer surgical treatment and reconstruction.

It is considered that in cases of facial injury the damage to the parotid duct is often overlooked or underestimated (8). In cases of oral cancer patients the surgical trauma of parotid duct can also be overlooked. We consider that in cases where buccal mucosa is resected the possibility of injury to the distal end of Stensen’s duct must be taken into consideration. In cases of reconstruction of oral cavity with free flaps or regional flaps the risk of obstruction and stenosis of distal end of parotid duct is increased. This results in extravasation of saliva in the soft tissues with subsequent formation of sialoceles, cutaneous fistulas or cysts of the salivary duct (8). These complications are difficult to treat, may become persistent and produce facial scarring. They can be avoided if the parotid duct injury is anticipated while planning oral mucosa resection as its proper intraoperative management is possible [see case 3]. If sialocele is taken into consideration as a possible postoperative complication it can be diagnosed rapidly and treated correctly avoiding its transformation into cutaneous fistula and damage to surgical reconstruction as the presence of saliva can cause wound breakdown reducing tensile strength of vicryl and chromic sutures (9).

The diagnosis of sialocele usually is not difficult if the history of possible parotid duct injury is taken into account. A sialocele presents as a swelling in the preauricular region which typically develops 8 to 14 days after the injury . In postoperative period it is frequently misdiagnosed as hematoma. On palpation the lesion is soft and mobile. No erythema of the skin, no fever, no supuration are observed. The patient can complaint of pressure sensation in the area (3). The amylase identification in the liquid obtained via nedle aspiration shows levels that exceed 10000 u/l (10). CT shows a single or multiloculated cystic lesion with smooth margins and internal density lower than that of surrounding tissues. The differential CT diagnosis includes retention cyst, sialodochitis, branchial cleft cyst and lymphoepitelial cyst (3). If sialocele is left untreated the swelling increases progressively and cutaneous fistula formation may produce.

The management of salivary duct injury and resultant sialocele remains controversial. Various conservative and surgical methods have been described. In general the management of the duct injury depends on the age of injury, site of injury and mechanism of injury. Three methods generally employed are based on primary repair of the duct, creation of oral fistula and suppression of parotid gland function (10). It is considered that parotid duct injuries are best repaired early at the time of injury (10). In case of oral cancer patients the whole distal end of Stensen’s duct is resected in block with tumour at the time of surgery, thus no direct repair is possible. The tecnique of diversion of the Stensen’s duct into the mouth could be useful at the time of resection of oral mucosa if the iatrogenic injury to its distal end has been anticipated at the time of surgical planning (4). Another tecnique applied intraoperatively is the creation of oral fistula [case 3].

If the salivary duct iatrogenic injury have been overlooked the sialocele may appear. The management of sialoceles includes multiple percutaneous needle aspirations, application of pressure dressings, use of antisialogogues drugs, injection of botulinum toxin (1). If these techniques don’t resolve the probleme then radiotherapy of salivary gland and surgical treatment have been proposed. Surgical options include ligation of parotid duct, primary repair of the duct, superficial or total parotidectomy/submaxilectomy. The repair of duct in cases of sialocele is considered to be challenging due to the presence of a fibrous scar over the area of injury and interposition of flap tissue.

The method of intraoral fistula surgical creation was reported by Morestin in 1917 for the management of parotid fistula after facial wounds. This method has been recently described for treatment of post-traumatic sialocele (9,3). There was no consensus about either the type of catheter to use [pigtail, silastic drains, Jackson Pratt, venous catheter] or the time to maintain it in the oral mucosa [four weeks, two months].

 The method of itraoral fistula creation has not yet been described for management of iatrogenic sialoceles. We successfully applied this method in one of our patients [case 2]. We consider this method to be physiologically based as once we observed spontaneous intraoral fistulization of iatrogenic sialocele [case 1].Besides it seems to be more comfortable for both the patient and the surgeon in comparison with multiple needle aspirations. This method is not challenged by presence of fibrous scar tissue or interposition of flaps as the method of duct repair or recanalization. It is technically simple and doesn’t require general anaesthesia.

## Conclusions

The iatrogenic injury to the distal end of the salivary duct is observed in oncological surgery when oral mucosa is to be resected. If not treated duct injury may result in such complications as sialocele, salivary fistula or ductal cysts. The iatrogenic sialoceles in oral cancer patients who underwent reconstruction of defect with free flap or regional flap has not yet been described. A number of techniques for management of salivary duct injuries have been proposed. If the injury to the duct is detected at the surgery or is anticipated in surgical plan then it can be resolved simultaneously with resection of tumour by placing intraoral drainage or duct reimplantation. We propose the method of intraoral drainage with over-the-needle cannula for iatrogenic sialoceles in oral cancer patients. We consider that it is important to anticipate the salivary duct injury in oncological patients as its intraoperative management is effective and prevents development of postoperative complications.
